# Zhuangzi and collaboration in animals: a critical conceptual analysis of shared intentionality

**DOI:** 10.3389/fpsyg.2023.1170358

**Published:** 2023-06-29

**Authors:** Dennis Papadopoulos

**Affiliations:** Unit of Ethics and Human-Animal Studies, Messerli Research Institute, University of Veterinary Medicine Vienna, Vienna, Austria

**Keywords:** shared intentionality, conceptual analysis, great apes evolution, cooperation, philosophy, Chinese philosophy, Zhuangzi

## Abstract

Shared intentionality is a specific form of shared agency where a group can be understood to have an intention. It has been conjectured that humans are better equipped for collaboration than other animals because humans but not other great apes share intentions. However, exporting shared intentionality from a debate about the ontology of mental state attributions like intentions to groups does not seamlessly lend itself to evolutionary science. To explore and de-center the implicit assumptions of Western conceptions of cooperation, I look at Zhuangzi’s philosophy of (in)action. This philosophy treats the actions of individuals as always a form of co-action alongside other agencies to whom one must adapt. Thinking of collaboration as a product of skillful co-action, not shared intention, sidesteps asking about cooperation in “kinds” or levels. Instead, it directs attention to the know-how and behavioral flexibility needed to make our constant coordination adaptive.

## 1. Introduction

Shared intentionality is often used to describe a uniquely human capacity for collaboration (Kaufmann, 2012; Engelmann and Tomasello, 2017; Tomasello, 2020). However, shared intentions are also used to describe even the most trivial instances of human cooperation. The sentence “we intend to go for a walk together” includes a shared intention (Gilbert, 1990); the fact that walking together is a cognitively undemanding coordination task does not affect the “shared” quality of our intention. Many non-human animals also walk together intentionally. The supposed uniqueness of shared intentionality hinges on the idea that the cognitive processes that underlie human cooperation, even in cases like walking together, are qualitatively different from the cognitive processes underlying similar behaviors in non-human animals. For example, if a human rides on a horse, both the human and horse are cooperating, but only the human can think of their joint action as a collaboration performed by an intersubjective “we.”

Some argue that there is good reason to doubt that there is such a qualitative difference here because nonhuman animals exhibit relevant intersubjective awareness through norms, social maintenance, and communication (Papadopoulos, 2021; Papadopoulos and Andrews, 2022; Sievers, 2022). Additionally, joint action might be explained more minimally than shared intentionality (Vesper et al., 2010; Butterfill, 2012; Pacherie, 2013; Blomberg, 2015; Salomone-Sehr, 2022). The whole discussion, however, is underwritten by rhetoric of collaboration as a tool to achieve one’s individual goals. This rhetoric highlights questions about how we might obtain the capacity to use such a tool.

This intuition, however, leans on an implicit Liberal social-ontological tradition that we may not intentionally endorse. I suggest we introduce classical Chinese philosophy to disrupt taken-for-granted intuitions and unseen (and likely unintended) rhetorical framing about the capacity to collaborate.

Classical Chinese philosophy of action (including early Confucian and Taoist thought) frames all action as co-action and explains that skillful co-action includes adapting our methods and goals to the larger whole around us (Valmisa, 2021). In this model of collaboration, we might collaborate, better or worse, with other individuals, our family, political leadership, or other animals. Collaboration applies to other sorts of agency as well, humans and animals also collaborate with incorporeal agents, Heaven and Earth, this is manifest in adapting to changing seasons, circumstances, and opportunities. Attributing agency widely transforms the mere coaction of bodies in space into a collaboration. This allows Chinese philosophers to describe general goods like a just state, content life, or moral conduct, as the product of skillful collaboration with dynamic agencies around you, corporeal or otherwise (Valmisa, 2021). In such an ontology, collaboration is not a tool to deploy but an inevitable part of the background of all action for social creatures embedded in a dynamic world.

We can look at coordination and collaboration as widespread phenomena where complex cognitive tools might sometimes help us collaborate better, but this “better” collaboration can be the result of pluralist and diverse mechanisms. Looking for skillful collaboration among the myriad forms of collaboration side-steps questions about differences in kind of cooperation, shifting us from a “stage by stage” view of the evolution of collaboration to a more fluid gradualist view where collaboration is skillful coaction with other agents. Whether it is appropriate to call that collaboration shared intentional or not is a separate question that may not depend on the cognitive capacities or know-how participants require. Looking for the behaviors associated with skillful collaboration may focus our attention on non-human animal-centred questions like what know-how, communication (Heesen et al., 2020, 2021a,b), patterns or norms (Andrews, 2020; Andrews et al., in preparation; Westra and Andrews, 2022), might facilitate collaboration.

The suggestion that skillful collaboration varies gradually in degrees rather than kind does not reject the central claim of the Vygotskian Intelligence Hypothesis, that human cognition even in early childhood shows signs of remarkably cooperative dispositions (Moll and Tomasello, 2007). After all, humans regularly cooperate with complete strangers and form projects and societies of enormous scale. However, even thinking in “better or worse” reduces the vibrant range of cognitive capacities that might support skillful collaborations across species. Describing collaborative skills opens up the possibility of attributing different skill sets and abilities to different species without trying to evaluate whether other species participate in a plausibly unique human form of collaboration.

To explain how skillful collaboration evolves, we may want to explore features of skillful collaboration identified in classical Chinese thought, and particularly in the Zhuangzi (an early Taoist text attributed to a sage of the same name). This requires recognizing that our goals are often not fixed, our understanding of others may be partial, and our processes of continuing to adjust to others blur the clean line between acting together and acting solitarily. Zhuangzi draws our attention to the problems of coordinating our actions in conditions of uncertainty with unknowable others. He suggests coordinating action by practicing behavioral flexibility regarding when we follow or choose not to follow established patterns of behavior. Finally, and perhaps most importantly, skillful coordination with unknowable others may require being very flexible about our goals, inhibiting our prior aims or behavior patterns to better align ourselves with our situations.

Shared intentionality, as cognitive quality sometimes present in collaborative behavior, does not neatly pick out, and might conflict with, the kind of exceptionally skillful degree, frequency, or scale of human collaboration. As a result, I suggest we can be agnostic about whether the uniquely human forms of collaboration form a distinct kind, like a “shared intention.” Instead, we might refocus on the many forms of know-how, communicative tools, and established behavior patterns that humans and other animals flexibly deploy or inhibit.

## 2. Unsuitability of “shared intentionality”

My first argument aims to shake our faith in the construct validity of shared intentionality as operationalized in comparative cognition research. The meta-cognitive processes that describe shared intentions may not be necessary for participation. As such, we may want to tease apart the ontological descriptions attributing mental states like intentions to groups from the cognitive processes that lead to that group action. This distinction will lead us through the historical background explaining how four peculiar and inessential behaviors—role-reversal, ongoing communication, commitment to complete an activity, and preferential partner-sharing—came to demarcate uniquely human collaboration from the mere cooperation of other species.

The concept of shared intentionality was popularized by John Searle (1990, 1995), who suggested that “we-intentions” can help explain the social structure under our shared institutions. He suggested concepts as diverse as baseball games, money, and language can be understood as shared expressions which often took the propositional form: we intend that some action or object be taken to have a specific meaning in a specific context. One element of special interest here is that individuals do not need to be able to articulate the rules they follow to assign context-specific meanings; rather, these rules describe our collective behavior. This leaves open questions about the structure; what exactly do individuals need to do or know for us to describe their behavior as part of a “we-intention?” Two theories about the supposedly minimal structure of small-scale “joint intentions” have been especially influential in the discourse around the evolution of shared intentionality: Michael Bratman’s “planning agent” and Margaret Gilbert’s “plural agent” accounts.

### 2.1. Planning agents, ongoing communication and role-reversal

Bratman (1992, 2009, 2018) suggests, very roughly, that joint intentions occur when individuals plan to achieve a shared goal through coordinated behavior and express a commitment to help others by performing part of the tasks required to obtain that goal. For example, two people decide to meet at the train station, which is a shared goal. To get there, they have a different set of tasks. But through planning, where both understand their distinct roles in the plan, they can meet at the right gate and time. The “plan” is the unifying feature coordinating each of their “sub-plans” or roles. The plan lets us correctly pick out that “they intend” as a shared unit and not merely that each of them has an intention (one to go to the train station by train and the other to walk to that same train station).

It’s important to note that this description is sufficient but not necessary (Bratman, 2018, p. 184). It is plausible that behavior is appropriately described in shared intentional terms without the participants thinking of themselves as engaging in a coordination task aimed at a shared goal; other heuristics might be employed to achieve the shared intention. Imagine the person traveling by train has a child. The child participates with the simple sub-plan of “follow my parent,” maybe with an understanding that eventually, they will meet someone familiar. It’s not clear that the child has to understand the entire plan to participate in the shared intention to meet. What is important for Bratman is that the shared intention is reducible to a system of individual intentions (sub-plans) which form a coherent and consistent plan.

If instead of a child, we imagine a pet dog on the trip, it becomes less clear if the dog’s nearly identical sub-plan “follow my human” is sufficient. We can exclude the dog in this case because it’s not clear that they know that their sub-plan “follow my human” meshes with the collective plan to “meet at the train station.” But the dog nonetheless expresses the know-how needed to participate. Shared intentions that include ostensibly demonstrable knowledge of others’ plans or intentions are distinct from know-how to participate. However, this distinction hinges on linguistic capabilities, not the nature of their agency or attentiveness to others (Sievers, 2022). If shared intentionality is meant to describe a certain sort of group, where all members are responsible for an intended action, then the ostensible communication and explicit contribution may be important. But if we want to explain how such behavior evolved, or how individuals come together to in-practice mesh their intentional behavior, then this account of shared intentionality may not be a useful conceptual tool.

Furthermore, it might miss the social cognition involved in the aspects of the shared travel in which the dog participates. Dogs can gaze-follow, and look back and forth to pick up ostensive cues about the local environment and where their humans are attending (Huber and Lonardo 2023). This allows the dog to coordinate action sufficient to meet the goals of sub-plans associated with travelling together. In this way, the apparent difference in the kind of cooperation being used is a product of a comparably less interesting difference in linguistic capacity.

Boesch (2002) introduced Bratman’s account to comparative cognition when he claimed that chimpanzees shared the intention to hunt monkeys, evidenced by the observation that in the Taï forest, chimpanzees adopted distinct roles. In Parallel, Tomasello et al. (2005a) proposed, also leaning on Bratman’s conception, that shared intentionality was uniquely human because of ontogenic markers associated with communication. Later Moll and Tomasello (2007) directly address this conflict with Boesch and suggest that the appearance of collaborative hunting is likely merely opportunistic behavior because chimpanzees lack key markers indicating they understand themselves as doing things together like role-reversal and ongoing communication.

The role-reversal argument suggested that if chimpanzees understood that they were playing different roles that fit together into a collective plan, performance in one role should improve performance in a coordinated role. This was found with children (Carpenter et al., 2005) but not with chimpanzees (Tomasello et al., 2005b). However, while learning from role-reversal tasks might indicate an understanding of doing something together, it does not seem necessary, and has been left out of some later discussions like Duguid and Melis’s (2020) reiteration of the other three capacities we’ll discuss.

Ongoing communication has been observed in children when given collaborative tasks, but even when chimpanzees communicate during collaborative tasks, they do not do so in an ongoing and persistent way (Melis and Tomasello, 2019). To better understand this, we might need to unpack what is being communicated, and then we might be able to see whether such ongoing communication is necessary for shared intentionality. For example, Heesen et al. (2020, 2021a,b) suggests that chimpanzees and bonobos use communicative cues to enter and exit from collaborative activities like grooming and play. In such cases, we might suggest the entrance and exit communication could be sufficient. More to the point, even if some communication is required for a shared intention, why must it be ongoing? Humans often collaborate with minimal communication. On Bratman’s model, we can even infer what role we ought to play from an understanding of the shared goals and tasks; virtually no actual communication is required.

### 2.2. Plural subjects, completing tasks and preferential sharing

To clearly articulate the implications of the human uniqueness of shared intentions, Tomasello (2016) suggested that social norms and morality are products of humanity’s unique shared intentionality. This leans on Gilbert’s conception that norms and obligations facilitate collaboration. Gilbert (1990, 2009, 2018) offers a compelling alternative theory of shared intentionality. She suggests that in shared intentions, the subject with the intention is a “plural subject” and that subject’s intentions need not reduce to individual intentions. However, she suggests that in a shared intention, we should expect that a norm is created such that individuals who fail to do their part are in a position to be reprimanded or otherwise encouraged to conform. While Gilbert focuses on negative reinforcement or “rebuke” in these cases, various systems of social maintenance (encouragement, positive affiliation, social hierarchy, etc.) can shape the behavior of individuals, human or animal, in such a way that it reinforces a cooperative pattern of behavior (Papadopoulos and Andrews, 2022; Westra and Andrews, 2022).

For example, if two individuals are walking together, and one spontaneously stops, that raises alarm. It’s deviating from the intention, and the person they are walking with is in a position to inquire or complain, “hey, why did you stop?” or “well, come on then!” This shows that walking together might differ from merely walking beside someone because it involves this normative element.

From this account, Tomasello (2016, 2020) and Duguid and Melis (2020) argue that because children, but not chimpanzees, complain after interrupted cooperative play (Warneken et al., 2006), children but not chimpanzees are thinking of their partners as obliged to complete the task together. Closely related to this understanding, they also argue that if there is an understanding of an obligation to complete a task, there also ought to be a partial and preferential sharing of goods among collaborators. That is, partners should share more with each other than bystanders or social affiliates. While we observe food sharing among great apes (Fruth and Hohmann, 2018; van Leeuwen et al., 2021), we do not observe them preferring to share with collaborators (Melis et al., 2011; John et al., 2019), but we do observe such preferential treatment of partners in children (Greenberg et al., 2010; Hamann et al., 2014).

However, we have reason to doubt that either of these criteria, continuing activities to completion or preferential sharing, accurately indicates a human-unique kind of collaboration. First of all, the social maintenance required to create conformity can be produced in many ways, not just rebuke, and there may be other explanations for chimpanzees not reinitiating play in an experimental setting (Papadopoulos and Andrews, 2022). Also, Heesen et al. (2021a) suggest that great apes reinitiate grooming behavior after the interruption. Expressive cues indicate the beginnings and ends of the cooperative activity. So “completing” the activity might overstate the commitment to a goal required for interruptable cooperative activities, and expressive cues or tolerance for interruption might need to be added to our models of what cooperative activity looks like.

Second, the implication that sharing norms should follow preferentially sharing with collaborators might overstate a sense of fairness in the cooperative activity. Gilbert (2020) has explicitly clarified that her view does not entail any norms of fairness. Sharing occurs often, and individuals who share might be more approachable in the context of both chimpanzees (Nishida et al., 1992) and human infants (Geraci et al., 2022). But within-group sharing norms might follow any number of models.

The sharing norms of adult human groups vary widely and may not closely rely on mutualistic collaboration or preferential sharing. Among the indigenous Siberian community of the Taimyr Peninsula, sharing with others according to the rule “never return a bag empty” was a cornerstone of a gift economy that shared, among other things, meat from hunting. Being given gifts correlates with participation in the gift economy by not returning bags empty; but is insensitive to the value of gifts given. In this way, in-group membership, marked by participation in general sharing norms, predicts sharing, not whether or not individuals collaborated (Ziker et al., 2016). Meat is not just shared with other hunters, or even with their families, but instead is distributed widely through the community. This is just one way to resolve distribution problems. Among duck hunters in Minnesota, it is important that you do not shoot at waterfowl near another blind or decoy; this allows each hunter to collect their share (Fix, 2021). Here not-sharing is the norm that leads to an appropriate and equitable distribution.

The supposition that children understand their collaboration in a distinctively human way because they prefer to share rewards with collaborators (playmates) more than others (Greenberg et al., 2010; Hamann et al., 2014), fails to recognize the range of variable ways humans share. The fact that chimpanzees share food with neighbours and social affiliates more than collaborators (Melis et al., 2011; John et al., 2019) tells us something about their collective behavior patterns (maybe even social norms), not how they think of their collaborators.

Rather than looking for reward distributions that incentivize collaboration, we may want to focus on a sense of “we” or social “bonding.” In a virtual setting, where adult humans were allowed to collaborate to hunt one of many targets, models of collaborative hunting behavior that relied on an imagined sense of “we” better described human behavior than models that relied on shared rewards (Tang et al., 2022). In such situations, it would be odd to say those humans did not collaborate, so this indicator of collaboration is also unnecessary.

Here we see the central problem with trying to attribute clear indicators of a uniquely human “kind” of collaboration. The theories about attribution of intentions to groups assume capable agents and are trying to differentiate when agents capable of shared intentions do them and when they do not. The flags that indicate this in human groups might include sharing with collaborators such that if several collaborators share or insist on completing a task they probably thought of each other as collaborating. This does imply that the lack of such features indicates a lack of the capacity for collaborating. The debate that Gilbert (2009) and Bratman (2009) engage in focuses on differences between their theory of what makes a joint action qualify as a shared intention. Their aim to explain how or why group behavior carries intentional features. They do not debate with each other about the extension of which cooperative behaviors count.

### 2.3. More restrictive accounts of shared intentionality

Shared intentionality is not always used so broadly; sometimes, “shared intentionality” distinguishes a type of metacognitive sense of intersubjectivity captured in language and self-report. In these cases, we might think of shared intentions as capturing a qualitatively interesting kind of behavior, but then we will want to exclude from this set some of the examples used in the philosophical literature. For example, in the ontogenic development of speech capacities, we notice developmental differences between the use of intersubjective language about “we” and earlier developing understandings about “you” and “I” even across languages (Tantucci, 2020; Tantucci and Wang, 2020). But such a difference in language use may not tell us much about the embodied phenomena of understanding oneself as a “we” (Ratcliffe, 2005; Zahavi, 2015).

We also see “shared intentions” used to identify different ways that individuals conceptualize their participation in collaboration, such that their capacity to collaborate is not in question. Sometimes autistic people or people with schizophrenia do not report sharing intentions in the sense that “(1) I am aware that you have a mental state, which qualifies as an intention (“mind reading”), and (2) this intention of yours figures in my pool of motivations in a particular way” (Salice and Henriksen, 2021, p. 3). While such self-reports of the explicit understanding of how one’s intentions may or may not mesh with the intentions of others can indicate an area of difficulty, that experience does not imply that these people cannot collaborate or that their struggles would be similar to the barriers to participation that might exclude great apes from shared intentions. So while “shared intentions” are used in many cases, from ontology to psychiatry, the term refers to a different set of practical indicators. Both the underlying mechanisms and the extension of exemplar behavior vary widely across disciplines.

Collaboration, intersubjective language, and self-report all use “shared intentions” but pick out different sets of behaviors. The view that features like role reversal, ongoing communication, insistence on completion, and preferential sharing with collaborators, suggests a unique kind (level, or tier) of sophisticated collaboration exaggerates the abstract thinking humans use to collaborate. Instead of seeing these four features as necessary capacities, we may want to reframe them as intuitive signs. The intuitions at stake here may stem from an implicit understanding of others as liberal subjects, who agree, through an implicit social contract, to live with others. When this conception of individuals is in the background it provides a scaffold for us to conceptualize collaboration as a miniature social contract, with commitments to help, obligations to fulfil, and shared responsibility for the group’s intentions. And this way of thinking is sometimes appropriate when we want to attribute responsibility or ostensively communicated decisions to groups like corporations (Salomone-Sehr, 2022) or juries (Pettit, 2010). However, we may want to be cautious about the rhetorical invocations of liberal subjects when we use terms like “communication,” “commitment,” and “obligation”; or of individualism more generally when we think about collective action in terms of individual “roles” or “preferences.” This rhetoric sets up a narrative of collaboration as a product of individuals’ roles, preferences, and commitments, where society, or collaboration, is a way to satisfy those ends. In this liberal social contract narrative, coordinated effort lifts us from lives which otherwise might be “solitary, poor, nasty, brutish, and short” (Hobbes, 1651/1996, i. xiii. 9).

We might be further concerned by the contemporary WEIRD (Western, Educated, Industrialized, Rich, and Democratic) presumptions implicit in this focus on the liberal subject. Keller (2012) suggests that highly educated Western people socialize children in ways that aim to develop what she calls Action Autonomy which is:

An individual’s capacity to act in a responsible and self-controlled way with respect to fulfilling responsibilities and obligations. This capacity comprises the planning and the performance of an action,^1^ which is under the control of the individual (Keller, 2012, p. 14).

But outside of WEIRD contexts, children are often socialized in ways that emphasize responsibility, and contribution to the family, or larger community. In place of Action Autonomy she charactizes these systems of socializing children as aimed at relatedness but specifies that “individuals can be in control of actions and situations without reference to their inner state of mind concerning themselves or others” (Keller, 2012, p. 14). The value of Action Autonomy and relatedness vary dramatically as do the strategies for developing it. Whether we focus on planning and inner mental states; contributions to the community through alloparenting at a young age, helping with chores; or respect for elders and self-control, does not undermine the importance of being in control of situations. However, that control can be developed in a wide variety of ways and capable children ought not to be evaluated on their action autonomy alone. I suggest there is a clear analog to the evolution literature. When we look for a liberal subject, with a well-developed Action Autonomy, we end up looking for one of many sufficient forms of competence, but risk mistaking that for a necessary feature of human evolution.

Social contract narratives might mislead us when we slip from discussing socio-political ontology to the evolution of cognitive capacities for collaboration. One version of this slippage occurs when Tomasello (2022) suggests vertebrates have goal-directed but not a properly intentional agency, mammals developed intentions as such, and apes develop rational agency. Equipped with rational and intentional agency, early human ancestors could think through the complex and abstract representations needed to mesh their sub-plans and share intentions. This reserves a special place for humans as the “political” animal uniquely equipped for sophisticated collaboration. This aligns well with the original human uniqueness claim that humans have “a species-unique motivation to share emotions, experience, and activities with other persons” (Tomasello et al., 2005a, p. 1).

It is a bit difficult to reconcile this narrative with observations of sociality that range widely across taxa. For example, snakes “actively seek social interaction, prefer to remain with larger aggregates, and associate nonrandomly with specific individuals or groups” (Skinner and Miller, 2020, p. 1). Perhaps snakes do this with only “goal-directed agency” and without intention, rationality, or shared intentions. However, implicit in the search for the most minimal account of the mechanisms of social behavior, such that goal-directed is more minimal and preferable to intentional or shared intentional, is the liberal subject. We presuppose that social behavior is the complex higher-order product of individual behavior—an agreement or concession we make to better achieve our individual goals. However, the know-how to participate in social activities might be surprisingly simple. Regardless of the mechanism at work, it seems that not just humans are motivated to share their experiences and activities.

## 3. Zhuangzi’s philosophy of action

Let me introduce a different narrative for connecting co-action to collaboration, one which de-centers Western intuitions about social behavior. In this case, I turn to classical Chinese philosophy, not because contemporary East-Asian perspectives provide an especially different conception of collaboration, but because an alternative narrative with new terms and structurally different rhetoric can reveal overlooked aspects of collaboration. I am not about to offer an in-principle incommensurable account collaboration that reveals the emptiness of shared intentionality. Instead, I want to shift the emphasis away from ideas and behaviors that might be overemphasized in taken-for-granted liberal rhetoric.

In Valmisa’s (2021) reading of classical Chinese philosophy, all action is understood as co-action across several traditions (including early Confucian and Taoist texts). Individuals are always acting alongside forces they cannot control—social circumstances, seasons, historical eras, and the wishes of others. For pragmatic reasons, humans and animals must coordinate behavior with the world around us, for example by changing our behavior with the passing seasons. This coordination includes being flexible about our goals and our methods. Part of this flexibility involves recognizing that we do not always know others, we may need to coordinate without knowing the aims of others, and we might need to adapt to (or join-in with) others whose intentions we cannot change. This conception of adapting to others minimizes the need for mind reading, so much so that we can imagine cooperating with patterns of nature or “fate.”

Thinking of our cooperation in terms of either meshing plans or abiding by obligations relies on information about the goals, plans, expectations, or obligations of ourselves and others in addition, perhaps, to their intentions. As such, we exclude from the outset the possibility of understanding behavioral flexibility in response to unknowable or unintentional changes in circumstance, season, or other animals whose plans are inaccessible.

Instead, Zhuangzi recommends adapting our goals and methods through inaction as a response to not-knowing. Zhuangzi is not proposing this as part of a philosophy of mind, and his mention of non-human animals is primarily metaphorical, nonetheless, his indirect commentary on socio-political and moral thought reveals a sense of collaboration aimed at harmonious living that is distinctly not about a liberal subject. His recommendation for inaction is not necessarily aimed at inactivity but inhibiting, ignoring, forgetting, or losing one’s individual goals and allowing the right course of action, with its often unknown aims, to arise from the confluence of opportunities around you. This self-inhibiting, opportunistic adapting to others will lead us to different expectations about the capacities that might underwrite humanity’s exceptional ability to collaborate.

Zhuangzi tells a story of a monkey keeper who tells the monkeys they will be fed three chestnuts in the morning and four in the evening. In response, they complain, so he suggests instead, they will be fed four in the morning and three in the evening. They are content with this (Zhuangzi, 1968). In this case, the monkey keeper “is flexible” in that he has “a commitment to walking not only one’s own path but the path of others.” (Valmisa, 2021, p. 41). By doing so, the keeper adapts to the monkey’s desires, which we see as beyond the keeper’s control. The keeper transforms antagonism into harmony by modifying their own behavior. The interesting highlight here is that Zhuangzi is attends to a particular sort of frustration the monkey keeper has. Zhuangzi often describes conforming with others as both necessary and frustrating. He says:

What is lowly and yet must be used: things. What is humble and yet must be relied upon: the people. What is irksome and must be attended to: affairs. What is sketchy and yet must be applied: the laws….What is confining but must be repeatedly practiced: ritual (Zhuangzi, as cited and translated in Nylan, 2017, p. 415).

Nylan (2017) argues that in this passage, Zhuangzi advocates for tolerance of others, their affairs, and rituals, but not because of a genuine belief, or sharing in their reasons. Zhuangzi generally treats human nature, like discontent monkeys, as a product of unreasoned inclination. Instead the skillful and adaptive actor participates in the unified behavior for the sake of harmony, in accordance with a content life of “free and easy wandering.” This other-directed concern composes one of Zhuangzi’s overarching concerns which is sometimes compared to a form of skepticism (Chung, 2017), but might be clarified as profound respect for difference, by treating others as far as possible, according to their own standards (Huang, 2010). For our purpose we might drop the moral account here and just suggest that a skilled collaborator can respect difference by not prioritizing their own aims, or conceptions of what would be good.

A second key theme is somewhat opposed to conformity. Zhuangzi often recommends living in spontaneous or creative way (Chung, 2022). This can seem like a contradiction encouraging conformity, but in both cases the recommendation includes “letting go” and shifting away from a “goal-directed, striving stance” (Wyatt, 1988, p. 97). For example, Zhuangzi tells a story about someone who makes gourds into containers for water. Given a huge gourd far too big to usefully hold water, he breaks the gourd. A wise man chastises him for narrowly viewing the giant gourd as a deficient water container; it was so large he could have cut it in half and made a boat! (Zhuangzi, 1968) Viewing the implicit possibilities without preconceived goals leads one to act in accordance with those things beyond our control that shape our circumstances.

Neither of these is a paradigmatic example of collaboration. But they highlight a new feature of how to collaborate that we do not find in the shared intentionality literature. Adapting means inhibiting or not-prioritizing your goals, methods, or preconceived notions in order to better align with others. It draws our attention to what is “not-done” in a case of collaboration; when we collaborate, we *do not* follow our inclinations unchecked. This not-doing creates space for being with others, not because we are committed to some outcome but because we are not committed to pursuing our individually decided courses of action.

In her discussion of adapting, Valmisa offers an explicitly cooperative coordination problem as her example of what co-action looks like. She explains that when we ride a horse, the “riding” is the product of coordinated action of the horse, human, and earth.

Riding only appears in the co-action between me and others, these others not limited to the horse but extending toward nonsubjective agents such as the terrain, the open space, the air. The event of riding necessitates multiple co-agencies in joint collaboration. [..] For instance, the horse regulates the force with which it pushes down the ground depending on the ground’s rigidity or softness; her muscles and breath send me messages of desire and capability to which my own muscles and breath unconsciously respond by tightening or loosening their pressure. Communication transcends our discrete bodies sending energy from one another to form the provisional third body which lies at the heart of riding (Valmisa, 2021, p. 178).

In this case, Valmisa is trying to explain how individuals adapt to the many intersecting forms of action beyond their control. She calls these many things fate (*ming*) and the practice of adapting to them through acting with them the “unifying pattern.” She suggests collaboration, like riding a horse, involves unifying one’s behavior with a larger pattern, including features out of our control. The horse, in this way, is both a literal example and a metaphor for Zhuangzi’s claim that he “rides the dao” (follows a unifying pattern) in all things.

## 4. Four indicators of collaboration—reimagined

We can think of collaboration as abiding by a unifying pattern where one suspends their own goals and inclinations to better adapt to the activities of others. With such a conception in mind, let us reconsider the four indicators of uniquely human shared intentions (1) learning through role-reversal, (2) ongoing communication, (3) insistence on task completion, and (4) preferential sharing among collaborators. For each of these we will consider additional and related indicators of skillful collaboration understood as a form of harmonizing with often unknowable but nevertheless dynamic circumstances of social life.

### 4.1. Other-directed attention

First, learning through role reversal and thinking of our plans as explicitly meshing focuses on explicit knowledge of another’s position as-if in some negotiation aimed at mutual benefit. However, we need not conceptualize the collaboration of horse and rider, or monkey and keeper, as knowing each other’s roles. They can still adapt to each through a sense of care and attentiveness that allows the other, thier roles, plans, and reasons, to be inaccessible. I cannot reverse roles with a horse and produce riding; our being unlike each other allows riding to emerge. Or, for the monkey keeper, the inaccessibility of the monkeys’ reasons is part of what prompts the adaptation; they are monkeys, and they will not be persuaded. Zhuangzi implies that humans are often the same way, so we had best just let go of our preconceived inclinations and adapt. Instead of looking for an abstract understanding of how plans or roles fit together to help us pursue shared goals, even where that goal might just be harmonious and peaceful coexistence.

The other-directed attentiveness implicit in learning through role reversal or meshing of plans treats the other as a rational subject with accessible reasons. But other-directed attentiveness can also manifest in forms of care for others that preserve a sense of radical difference. To find such behavior in nonhuman animals we should start by looking for care and other forms of collective engagement. And there is good reason to think care related to collaboration is found widely among animals. In some cases coperative care-giving is foundational for the development of valuable relationships or community-at large for example in the practice of parental care in a wide range of species (Wrage, 2022); alloparenting in social mammals (Lukas and Clutton-Brock, 2018), such as bats (McCracken and Gustin, 1991) and chimpanzees (Nishida, 1983); even birth assitants in chimpanzees (Hirata et al., 2011) and bats (Kunz et al., 1994). Further, if collaboration can be understood to include maintaining valuable relationships, then caring attention in reconciliatory (Webb, 2012) and consoling behavior (Goldsborough et al., 2020; Heesen et al., 2022) should also be included. These forms of other-directed attention offer building blocks for social life, and skillful attentiveness to others will allow individuals to maintain control, relationships, and harmony within a community without the need for knowing the plans and goals of others. The shared aspect of mental life is already present in embodied engagement.

The liberal-subject narrative haunts even conceiving of skillful attending. Bard et al. (2022) examined how cultural variation among humans and chimpanzees might affect how they direct attention. A certain sort of “triadic awareness” is common among primates (De Waal and Waal, 2007; Kubenova et al., 2017) but joint attention is sometimes used to pick out a supposedly uniquely human form of attention to others, which allows us to understand their plans and, therefore, to form shared intentions. This exclusively human might be better named “joint attention to mental states” (O’madagain and Tomasello, 2021). However, we might be concerned about how this overemphasizes WEIRD human narratives about child development. Bard et al. (2022) found joint attention is often defined by focusing on objects, explicit communication, and mental states or goals, and these definitions do not align closely with the socialization strategies of non-WEIRD communities (specifically Nso and Aka communities in Cameroon and Central African Republic). When indicators were adjusted to capture better the diversity of joint attentional engagement (joint engagement) among humans, they found chimpanzees perform similarly in joint engagement tasks. Decolonizing conceptions of attention involved focusing on interaction rather than object-directed play and embodied communication (including touch) rather than explicit verbal expressions. This suggests there is a wide range of skills we use in dynamic and shared activities, especially in parent–child relationships, that maintain coordination, and facilitate shared experiences.

### 4.2. Intimacy and embodied communication

The second indicator of shared intentionality was ongoing communication, but we have already suggested this conception is problematically fixated on explicit verbal communication about mental states. Instead of ongoing communication of that sort, we might reflect on the intimate feedback of touch found in Valmisa’s (2021) example of horse and rider. There bodies cue each other to respond, not just to the mental-states of one-another but to their embodied activity, emotionality, and the effect of the wider world (the earth under the horse’s hooves). Subtle forms of ongoing feedback, like touch, might give us one way of thinking about communication differently. Intimacy and physical closeness, are related to trust and care through tolerating each other’s touch (Monsó and Wrage, 2021) even mere proximity in walking together could contribute to reconciliation (Webb et al., 2017). Closeness does not just express mental states it shapes them, allowing for the maintainence of social relationships. Instead of taking a case like hunting as paradigmatic of collaboration, with shared plans and external objects, we might focus on something relationship-building like a hug. If we hug, it is not merely the case that you hugged, and I hugged, we coordinate and intended that hug together. Further, as soon as we touch, we are in ongoing communication.

To better understand cases of collaboration like this we might want to focus on overt cues that indicate when close joint engagement activity is proposed, and when it ends. Heesen et al. (2020, 2021a,b), suggests that in contexts of play or grooming, there are distinctive expressions that indicate a begining and end to the shared activity. This kind of communication may not be ongoing, but clearly indicates collaborative activity and even a joint commitment to engage.

Communicative cues associated with closeness also help coordinate group-action on a larger scale. For example, when baboons travel, proximity is still part of the shared embodiment of their space. As others gradually move more and more frequently, the group will conform to a unifying pattern where they must stop their independent foraging to stay with the group. They also do not just act automatically; they are selective, preferentially following elders (Strandburg-Peshkin et al., 2015). This does not mean they have ongoing explicit communication. However, they still attend to others leading to the provisional body of the troop, which decides collectively to move and does so through the ongoing subtle, sensual expression of travelling cues which allows them to participate in a unifying pattern, just like the horse and rider do through touch.

### 4.3. Conforming to the patterns of others

The third feature of shared intentionality we discussed was continuing a task to its completion. This can be indicated by a failure to exercise social maintenance like rebuke when collaboration breaks down. But it presumes a very distinctive goal, the sort of collaborative task that gets completed. In some situations achieving a reward is not the goal. Indeed Zhuangzi teaches us to be flexible with our goals so as not to miss opportunities to collaborate, as in the monkey keeper’s case of conforming with the monkeys. The frustration of the monkey keeper is not his attempt to understand the reasons or goals and to think through how to comply but rather how to inhibit the inclination to refuse to comply with their apparent nonsense.

Understanding collaboration as directed at a kind of harmony through inaction (and inhibition) might suggest doing things together and being motivated to share our experiences is an end itself, without the need for some further goal. Consider how chimpanzee females immigrating to a new group modify their nut-cracking behavior to adapt to others in the new group (Luncz et al., 2018). It’s not clear that this behavior is being socially maintained, they need not be ostracized, yet they will adopt the new behavior even if it is less efficient. This shows an interest in adapting, being part of the group and doing things the way they do, even if no one cares.

Applied to a case of collaboration, we might suggest that when someone fails to complete a task, stops participating, etc., exercising any form of social maintenance is not necessarily adaptive. Here we might say that human children that reinitiate cooperative play in a demanding way might even be less skilled collaborators because they are not adapting to others. They insist on things their way (like the monkeys who demand to feed more in the morning). Adapting to the group’s patterns of behavior or a partners’ behaviors can be other-directed, not goal-directed. This lets us be with others on their terms, like the monkey keeper who adapts to their monkeys.

### 4.4. Behavioral flexibility regarding established patterns

The fourth, and final, indicator of shared intentionality is preferential sharing between collaborators. I already suggested that it is both not a good example of Gilbert’s account of obligation and may not describe human behavior well. The central intuition is that collaboration often is “about achieving a goal” and therefore sharing the resulting fruit among collaborators indicates that they think of each other as working together. But Zhuangzi tells us that skillful collaboration often requires not having a set goal, and being radically flexible and open to new opportunities.

Let us reimagine this feature starting from the view that agents are already coordinating, but might do so in a more or less flexible way. In some cases where there is an established norm of sharing with collaborators, the lack of sharing seems plausibly uncooperative. The norm violator might be “using” others. But when there is no such norm, it might be odd to call not-sharing uncooperative. This reference to established patterns is especially important as we notice variations in helping and food-sharing behaviors among groups of great apes. When several groups were given access to a juice fountain that only worked if a conspecific pushed the button, van Leeuwen et al. (2021) noticed that some groups gregariously pushed buttons for others and some did not.

So where there are norms that guide group behavior, we might look to a couple of lessons from Zhuangzi. Adapting does not always mean following the norm; think of the gourd craftsman who should have made a boat with the giant gourd. Following or not-following the norms ought to align with other elements of unifying patterns, and the way to do that is to let go of one’s presuppositions. In a term, “behavioral flexibility” in contexts of established behavior patterns indicates skillful adaptation to circumstances. Humans are very flexible in our ability to follow new norms, meet new people, tolerate norm violations, or socially maintain behavior patterns in the face of norm violations. These skills do not indicate an individualistic autonomy and keen awareness of mental-state language, in the way “shared intentional” developmental narratives emphasize. Indeed those narratives are better associated with the socialization goals of WEIRD parents (Keller, 2012). Instead we might look for flexibility in the ability to inhibit, or let-go, of our preferences for the familiar and normal.

Consider the intercommunity foraging behavior of Bonobos. When foraging groups meet, it can be tense and stressful, especially for males. But in many cases, some females break down the tension by recognizing members of the group they may have immigrated from. The males might protest but eventually give in and tolerate these strangers. They forage together and then depart (Itani, 1990; Furuichi, 2011). The interesting feature is not whether or not their collaborative foraging qualifies as a shared intention. This case is peculiar because bonobos are uncommonly willing to adapt to a situation and uncommonly flexible about group membership (Samuni et al., 2022). This kind of behavioral flexibility suggests the skilled application of knowing when and how to collaborate by inhibiting one’s inclinations (in this case, inhibiting intolerance of strangers).

## 5. Discussion

Focusing on how animals collaborate, maintain contact, and conform or violate established patterns offers a way of exploring skillful collaboration that side-steps shared intentionality. We can reimagine collaboration as the product of adapting to others; this adapting is a skillful exercise of the co-action in which we are always engaged. In such cases, we would not look for indicators of a peculiar, uniquely human kind of collaboration, which does not necessarily involve understanding roles reversibly, ongoing communication, a commitment to obtain a goal for all involved, or preferential sharing of rewards with partners. Instead, we would look for other-directed care, unifying patterns of behavior where group members attend to one another (especially if it involves touch or moving as a group), a willingness or interest in adapting to others as a group or as partners, and a behaviorally flexible engagement with established patterns of behavior. These are, like the prior four, just indicators that adapting to others in social contexts might be happening; they are not necessary conditions. But since I am describing a skill that applies to collaboration and other contexts, and I am not trying to articulate a distinctive kind of collaboration, the indicators need not be necessary.

The questions these indicators set up is not who belongs in the shared-intention club, but rather how is it that humans are so effective at collaborating? And how might other animals deploy different skills in their collaborations and cultures? Zhuangzi offers an alternative ontology to shared intentionality but not an incompatible one. There is nothing about adapting, according to Zhuangzi (or Valmisa, 2021), that suggests we should (or should not) ascribe intentions to groups. It just offers a different narrative, one that might help us challenge and de-center Western presumptions of the liberal subject. Changing the narrative also adjusts our questions. Exploring further and critically, the suite of tools we use to interact (Heesen and Fröhlich, 2022) without demarcating a kind of collaboration might help us identify which important practices nonhuman animals might not be able to exercise effectively. We might want to further explore the systems of social maintenance and norms that individuals flexibly engage with or violate (Andrews et al., in preparation) without presupposing that norms which seem beneficial, like preferential sharing with partners, are needed to motivate norm-following. The features of collaboration which we should remember are the caring, embodied, intimate, and flexible skills that allow us to let go of our inclinations and adapt to others, as we do in riding or hugging.

## Author contributions

The author confirms being the sole contributor of this work and has approved it for publication.

## Funding

This work was funded by the “Morality in Animals” project with the Austrian Science Fund (Project Number P31466-G32).

## Conflict of interest

The author declares that the research was conducted in the absence of any commercial or financial relationships that could be construed as a potential conflict of interest.

## Publisher’s note

All claims expressed in this article are solely those of the authors and do not necessarily represent those of their affiliated organizations, or those of the publisher, the editors and the reviewers. Any product that may be evaluated in this article, or claim that may be made by its manufacturer, is not guaranteed or endorsed by the publisher.

## Footnotes

^1^Here Keller cites: “Heckhausen, J., and Heckhausen, H. (Eds.). (2006). *Motivation und Handeln [Motivation and action]*. Heidelberg, Germany: Springer Medizin.”

## References

[ref1] Andrews K. (2020). Naïve normativity: the social foundation of moral cognition. J. Am. Philos. Assoc. 6, 36–56. doi: 10.1017/apa.2019.30

[ref2] Bard K. Keller H. Ross K. Hewlett B. Butler L. Boysen S.. (2022). Joint attention in human and chimpanzee infants in varied socio-ecological contexts. Monogr. Soc. Res. Child Dev. 86, 7–217. doi: 10.1111/mono.12435, PMID: 35355281

[ref3] Blomberg O. (2015). “An account of Boeschian cooperative behaviour” in Collective agency and cooperation in natural and artificial systems: explanation, implementation and simulation. ed. Misselhorn C. (Heidelberg: Springer), 169–184.

[ref4] Boesch C. (2002). Cooperative hunting roles among tai chimpanzees. Hum. Nat. 13, 27–46. doi: 10.1007/s12110-002-1013-6, PMID: 26192594

[ref5] Bratman M. E. (1992). Shared cooperative activity. Philos. Rev. 101, 327–341. doi: 10.2307/2185537

[ref6] Bratman M. E. (2009). Modest sociality and the distinctiveness of intention. Philos. Stud. 144, 149–165. doi: 10.1007/s11098-009-9375-9

[ref7] Bratman M. E. (2018). Planning, time, and self-governance: essays in practical rationality. New York Oxford University Press

[ref8] Butterfill S. (2012). Joint action and development. Philos. Q. 62, 23–47. doi: 10.1111/j.1467-9213.2011.00005.x

[ref9] Carpenter M. Tomasello M. Striano T. (2005). Role reversal imitation and language in typically developing infants and children with autism. Infancy 8, 253–278. doi: 10.1207/s15327078in0803_4

[ref10] Chung J. (2017). Taking skepticism seriously: how the Zhuang-Zi can inform contemporary epistemology. Comp. Philos. 8:5. doi: 10.31979/2151-6014(2017).080205

[ref11] Chung J. (2022). Creativity and yóu: the Zhuāngzǐ and scientific inquiry. Eur. J. Philos. Sci. 12, 1–26. doi: 10.1007/s13194-022-00448-y

[ref12] De Waal F. Waal F. B. (2007). Chimpanzee politics: Power and sex among apes. Baltimore John Hopkins University Press

[ref13] Duguid S. Melis A. P. (2020). How animals collaborate: underlying proximate mechanisms. Wiley Interdiscip. Rev. Cogn. Sci. 11:e1529. doi: 10.1002/wcs.1529, PMID: 32342659

[ref14] Engelmann J. M. Tomasello M. (2017). The middle step: joint intentionality as a human-unique form of second-personal engagement. In The Routledge handbook of collective intentionality Jankovic M. Ludwig K. (pp. 433–446). New York, NY Routledge

[ref15] Fix C. (2021). The unspoken etiquette of duck hunting. Available at: https://projectupland.com/waterfowl-hunting-2/the-unspoken-etiquette-of-duck-hunting-2/

[ref16] Fruth B. Hohmann G. (2018). Food sharing across borders: first observation of in-tercommunity meat sharing by bonobos at Lui Kotale, DRC. Hum. Nat. 29, 91–103. doi: 10.1007/s12110-018-9311-9, PMID: 29619667 PMC5942352

[ref17] Furuichi T. (2011). Female contributions to the peaceful nature of bonobo society. Evol. Anthrop. 20, 131–142. doi: 10.1002/evan.20308 22038769

[ref18] Geraci A. Simion F. Surian L. (2022). Infants’ intention-based evaluations of distributive actions. J. Exp. Child Psychol. 220:105429. doi: 10.1016/j.jecp.2022.105429, PMID: 35421629

[ref19] Gilbert M. (1990). Walking together: a paradigmatic social phenomenon. Midwest Stud. Philos. 15, 1–14. doi: 10.1111/j.1475-4975.1990.tb00202.x

[ref20] Gilbert M. (2009). Shared intention and personal intentions. Philos. Stud. 144, 167–187. doi: 10.1007/s11098-009-9372-z

[ref21] Gilbert M. (2018). Remarks on joint commitment and its relation to moral thinking. Philos. Psychol. 31, 755–766. doi: 10.1080/09515089.2018.1486611

[ref22] Gilbert M. (2020). Shared intentionality, joint commitment, and directed obligation. Behav. Brain Sci. 43, e71–e28. doi: 10.1017/S0140525X19002619, PMID: 32349830

[ref23] Goldsborough Z. van Leeuwen E. J. Kolff K. W. de Waal F. B. Webb C. E. (2020). Do chimpanzees (*Pan troglodytes*) console a bereaved mother? Primates 61, 93–102. doi: 10.1007/s10329-019-00752-x, PMID: 31485897 PMC6971188

[ref24] Greenberg J. Hamann K. Warneken F. Tomasello M. (2010). Chimpanzee helping in collaborative and noncollaborative contexts. Anim. Behav. 80, 873–880. doi: 10.1016/j.anbehav.2010.08.008

[ref25] Hamann K. Bender J. Tomasello M. (2014). Meritocratic sharing is based on collaboration in 3-year-olds. Dev. Psychol. 50, 121–128. doi: 10.1037/a0032965, PMID: 23688170

[ref26] Heesen R. Austry D. A. Upton Z. Clay Z. (2022). Flexible signalling strategies by victims mediate post-conflict interactions in bonobos. Philos. Trans. R. Soc. B Biol. Sci. 377:20210310. doi: 10.1098/rstb.2021.0310, PMID: 35934966 PMC9358318

[ref27] Heesen R. Bangerter A. Zuberbühler K. Iglesias K. Neumann C. Pajot A.. (2021a). Assessing joint commitment as a process in great apes. iScience 24:102872. doi: 10.1016/j.isci.2021.102872, PMID: 34471860 PMC8390869

[ref28] Heesen R. Bangerter A. Zuberbühler K. Rossano F. Iglesias K. Guéry J.. (2020). Bonobos engage in joint commitment. Science. Advances 6:eabcd1306. doi: 10.1126/sciadv.abd1306, PMID: 33355132 PMC11206216

[ref29] Heesen R. Fröhlich M. (2022). Revisiting the human ‘interaction engine’: comparative approaches to social action coordination. Philos. Trans. R. Soc. B 377:20210092. doi: 10.1098/rstb.2021.0092, PMID: 35876207 PMC9315451

[ref30] Heesen R. Zuberbühler K. Bangerter A. Iglesias K. Rossano F. Pajot A. (2021b). Evidence of joint commitment in great apes’ natural joint actions. R. Soc. Open Sci. 8:211121. doi: 10.1098/rsos.211121, PMID: 34909217 PMC8652280

[ref31] Hirata S. Fuwa K. Sugama K. Kusunoki K. Takeshita H. (2011). Mechanism of birth in chimpanzees: humans are not unique among primates. Biol. Lett. 7, 686–688. doi: 10.1098/rsbl.2011.0214, PMID: 21508028 PMC3169058

[ref301] Hobbes T. (1651/1996). Leviathan. Oxford, UK: Oxford University Press.

[ref32] Huang Y. (2010). The ethics of difference in the Zhuangzi. J. Am. Acad. Relig. 78, 65–99. doi: 10.1093/jaarel/lfp082

[ref302] Huber L. Lonardo L. (2023). Canine perspective-taking. Anim. Cogn. 26, 275–298. doi: 10.1007/s10071-022-01736-z, PMID: 36629935 PMC9877093

[ref33] Itani G. (1990). Relations between unit-groups of bonobos at Wamba, Zaire: encounters and temporary fusions. Afr. Study Monogr. 11, 153–186. doi: 10.14989/68066

[ref34] John M. Duguid S. Tomasello M. Melis A. (2019). How chimpanzees (*Pan troglodytes*) share the spoils with collaborators and bystanders. PLoS One 14:e0222795. doi: 10.1371/journal.pone.0222795, PMID: 31545837 PMC6756536

[ref35] Kaufmann A. (2012). Collective intentionality: a human–not a monkey–business. Phenomenol. Mind 2, 98–105. doi: 10.13128/Phe_Mi-19629

[ref36] Keller H. (2012). Autonomy and relatedness revisited: cultural manifestations of universal human needs. Child Dev. Perspect. 6, 12–18. doi: 10.1111/j.1750-8606.2011.00208.x

[ref37] Kubenova B. Konecna M. Majolo B. Smilauer P. Ostner J. Schülke O. (2017). Triadic awareness predicts partner choice in male–infant–male interactions in Barbary macaques. Anim. Cogn. 20, 221–232. doi: 10.1007/s10071-016-1041-y, PMID: 27734208

[ref38] Kunz T. H. Allgaier A. L. Seyjagat J. Caligiuri R. (1994). Allomaternal care: helper-assisted birth in the Rodrigues fruit bat, *Pteropus rodricensis* (Chiroptera: Pteropodidae). J. Zool. 232, 691–700. doi: 10.1111/j.1469-7998.1994.tb04622.x

[ref39] Lukas D. Clutton-Brock T. (2018). Social complexity and kinship in animal societies. Ecol. Lett. 21, 1129–1134. doi: 10.1111/ele.13079, PMID: 29797749

[ref40] Luncz L. Sirianni G. Mundry R. Boesch C. (2018). Costly culture: differences in nut-cracking efficiency between wild chimpanzee groups. Anim. Behav. 137, 63–73. doi: 10.1016/j.anbehav.2017.12.017

[ref41] McCracken G. F. Gustin M. K. (1991). Nursing behavior in Mexican free-tailed bat maternity colonies. Ethology 89, 305–321. doi: 10.1111/j.1439-0310.1991.tb00376.x

[ref42] Melis A. P. Schneider A. C. Tomasello M. (2011). Chimpanzees, *Pan troglodytes*, share food in the same way after collaborative and individual food acquisition. Anim. Behav. 82, 485–493. doi: 10.1016/j.anbehav.2011.05.024

[ref43] Melis A. Tomasello M. (2019). Chimpanzees (*Pan troglodytes*) coordinate by communicating in a collaborative problem-solving task. Proc. R. Soc. B 286:20190408. doi: 10.1098/rspb.2019.0408, PMID: 30991932 PMC6501936

[ref44] Moll H. Tomasello M. (2007). Cooperation and human cognition: the Vygotskian intelligence hypothesis. Philos. Trans. R. Soc. B Biol. Sci. 362, 639–648. doi: 10.1098/rstb.2006.2000, PMID: 17296598 PMC2346522

[ref45] Monsó S. Wrage B. (2021). Tactful animals: how the study of touch can inform the animal morality debate. Philos. Psychol. 34, 1–27. doi: 10.1080/09515089.2020.1859100

[ref46] Nishida T. (1983). Alloparental behavior in wild chimpanzees of the Mahale Mountains, Tanzania. Folia Primatol. 41, 1–33. doi: 10.1159/000156117

[ref47] Nishida T. Hasegawa T. Hayaki H. Takahata Y. Uehara S. (1992). Meat sharing as a coalition strategy by an alpha male chimpanzee. Top. Primatol. 1, 159–174. doi: 10.1007/0-306-47461-1_11

[ref48] Nylan M. (2017). Zhuangzi: closet Confucian? Eur. J. Polit. Theo. 16, 411–429. doi: 10.1177/1474885117702793

[ref306] O’Madagain C. Tomasello M. (2021). Joint attention to mental content and the social origin of reasoning. Synthese 198, 4057–4078. doi: 10.1007/s11229-019-02327-1

[ref307] Pacherie E. (2013). Intentional joint agency: shared intention lite. Synthese 190, 1817–1839. doi: 10.1007/s11229-013-0263-7

[ref49] Papadopoulos D. (2021). Shared intentionality in nonhuman great apes: a normative model. Rev. Philos. Psychol. doi: 10.1007/s13164-021-00594-x

[ref50] Papadopoulos D. Andrews K. (2022). How social maintenance supports shared agency in humans and other animals. HUMANA.MENTE J. Philos. Stud. 15, 205–223.

[ref51] Pettit P. (2010). “Groups with minds of their own” in Social epistemology: essential readings. eds. Goldman A. Whitcomb D. (New York: Oxford University Press), 167–193.

[ref52] Ratcliffe M. (2005). Folk psychology and the biological basis of intersubjectivity. R. Inst. Philos. Suppl. 56, 211–233. doi: 10.1017/S1358246100008857

[ref53] Salice A. Henriksen M. G. (2021). Disturbances of shared intentionality in schizophrenia and autism. Front. Psych. 11:570597. doi: 10.1007/s10539-022-09857-y, PMID: 33643078 PMC7902514

[ref54] Salomone-Sehr J. (2022). Cooperation: with or without shared intentions. Ethics 132, 414–444. doi: 10.1086/716877

[ref55] Samuni L. Langergraber K. E. Surbeck M. H. (2022). Characterization of Pan social systems reveals in-group/out-group distinction and out-group tolerance in bonobos. Proc. Natl. Acad. Sci. U. S. A. 119:e2201122119. doi: 10.1073/pnas.2201122119 , PMID: 35727986 PMC9245655

[ref56] Searle J. R. (1990). “Collective intentions and actions” in Intentions in communication. eds. Cohen P. R. Morgan J. Pollack M. (Cambridge, MA: MIT Press), 401–415.

[ref57] Searle J. (1995). The construction of social reality. New York, NY: Simon and Schuster.

[ref58] Sievers C. (2022). Interaction and ostension: the myth of 4th-order intentionality. Philos. Trans. R. Soc. B 377:20210105. doi: 10.1098/rstb.2021.0105, PMID: 35876199 PMC9310185

[ref303] Skinner M. Miller N. (2020). Aggregation and social interaction in garter snakes (*Thamnophis sirtalis sirtalis*). Behav. Ecol. Sociobiol. 74:51. doi: 10.1007/s00265-020-2827-0, PMID: 33643078

[ref59] Strandburg-Peshkin A. Farine D. R. Couzin I. D. Crofoot M. C. (2015). Shared decision-making drives collective movement in wild baboons. Science 348, 1358–1361. doi: 10.1126/science.aaa5099, PMID: 26089514 PMC4801504

[ref60] Tang N. Gong S. Zhao M. Gu C. Zhou J. Shen M.. (2022). Exploring an imagined “we” in human collective hunting: joint commitment within shared intentionality. Available at: https://escholarship.org/uc/item/3wj722pb

[ref61] Tantucci V. (2020). From co-actionality to extended intersubjectivity: drawing on language change and ontogenetic development. Appl. Linguis. 41, 185–214. doi: 10.1093/applin/amy050

[ref62] Tantucci V. Wang A. (2020). From co-actions to intersubjectivity throughout Chinese ontogeny: a usage-based analysis of knowledge ascription and expected agreement. J. Pragmat. 167, 98–115. doi: 10.1016/j.pragma.2020.05.011

[ref63] Tomasello M. (2016). A natural history of human morality. Cambridge, MA: Harvard University Press

[ref64] Tomasello M. (2020). The moral psychology of obligation Shared Intentionality, joint commitment, and directed obligation. Behav. Brain Sci. 43:e56. doi: 10.1017/S0140525X19001742 32349830

[ref304] Tomasello M. (2022). The Evolution of Agency: Behavioral Organization from Lizards to Humans. Cambridge, MA: MIT Press.

[ref65] Tomasello M. Carpenter M. Call J. Behne T. Moll H. (2005a). Understanding and sharing intentions: the origins of cultural cognition. Behav. Brain Sci. 28, 675–691. doi: 10.1017/S0140525X05000129, PMID: 16262930

[ref66] Tomasello M. Carpenter M. Hobson R. P. (2005b). The emergence of social cognition in three young chimpanzees. Monogr. Soc. Res. Child Dev. 70, i–152. 10.1111/j.1540-5834.2005.00324.x 16156847

[ref67] Valmisa M. (2021). Adapting: a Chinese philosophy of action. New York Oxford University Press

[ref68] van Leeuwen E. J. DeTroy S. E. Kaufhold S. P. Dubois C. Schütte S. Call J.. (2021). Chimpanzees behave prosocially in a group-specific manner. Sci. Adv. 7:eabc7982. doi: 10.1126/sciadv.abc7982 33627415 PMC7904267

[ref69] Vesper C. Butterfill S. Knoblich G. Sebanz N. (2010). A minimal architecture for joint action. Neural Netw. 23, 998–1003. doi: 10.1016/j.neunet.2010.06.002, PMID: 20598504

[ref70] Warneken F. Chen F. Tomasello M. (2006). Cooperative activities in young children and chimpanzees. Child Dev. 77, 640–663. doi: 10.1111/j.1467-8624.2006.00895.x, PMID: 16686793

[ref71] Webb C. (2012). Motivations for reconciliation: regulatory mode, individual differences, and evolutionary considerations. Available at: https://ssrn.com/abstract=2086538

[ref305] Webb C. E. Romero T. Franks B. de Waal F. (2017). Long-term consistency in chimpanzee consolation behaviour reflects empathetic personalities. Nat. Commun. 8:292. doi: 10.1038/s41467-017-00360-7 28819207 PMC5561193

[ref72] Westra E. Andrews K. (2022). A new framework for the psychology of norms. Biol. Philos. 37. doi: 10.1007/s10539-022-09871-0

[ref73] Wrage B. (2022). Caring animals and care ethics. Biol. Philos. 37:18. doi: 10.1007/s10539-022-09857-y, PMID: 35637869 PMC9135829

[ref74] Wyatt T. (1988). Spontaneity and naturalness. J. Humanist. Psychol. 28, 97–107. doi: 10.1177/0022167888281005

[ref75] Zahavi D. (2015). Self and other: from pure ego to co-constituted we. Cont. Philos. Rev. 48, 143–160. doi: 10.1007/s11007-015-9328-2

[ref76] Zhuangzi (1968). The complete works of Chuang Tzu (B. Watson, Trans.) New York Columbia University Press

[ref77] Ziker J. Rasmussen J. Nolin D. (2016). Indigenous Siberians solve collective action problems through sharing and traditional knowledge. Sustain. Sci. 11, 45–55. doi: 10.1007/s11625-015-0293-9

